# Ethanol-Extracted Brazilian Propolis Exerts Protective Effects on Tumorigenesis in Wistar Hannover Rats

**DOI:** 10.1371/journal.pone.0158654

**Published:** 2016-07-08

**Authors:** Anna Kakehashi, Naomi Ishii, Masaki Fujioka, Kenichiro Doi, Min Gi, Hideki Wanibuchi

**Affiliations:** Department of Molecular Pathology, Osaka City University Graduate School of Medicine, 1-4-3 Asahi-machi, Abeno-Ku, Osaka 545–8585, Japan; IIT Research Institute, UNITED STATES

## Abstract

The present study was conducted over a course of 104 weeks to estimate the carcinogenicity of ethanol-extracted Brazilian green propolis (EEP). Groups of 50 male and 50 female Wistar Hannover rats, 6-week-old at commencement were exposed to EEP at doses of 0, 0.5 or 2.5% in the diet. Survival rates of 0.5% and 2.5% EEP-treated male and female rats, respectively, were significantly higher than those of respective control groups. Overall histopathological evaluation of neoplasms in rat tissues after 2 years showed no significant increase of tumors or preneoplastic lesions in any organ of animals administered EEP. Significantly lower incidences of pituitary tumors in 0.5% EEP male and 2.5% EEP female groups, malignant lymphoma/leukemia in both 2.5% EEP-treated males and females and total thyroid tumors in 0.5% EEP male group were found. Administration of EEP caused significant decreases of lymphoid hyperplasia of the thymus and lymph nodes in 2.5% EEP-treated rats, tubular cell hyperplasia of kidneys in all EEP groups, and cortical hyperplasia of adrenals in EEP-treated females. In the blood, significant reduction of neutrophils in all EEP-treated males and band neutrophils in 2.5% EEP-treated females was found indicating lower levels of inflammation. Total cholesterol and triglicerides levels were significantly lower in the blood of 2.5% EEP-treated female rats. In conclusion, under the conditions of the 2-year feeding experiment, EEP was not carcinogenic, did not induce significant histopathological changes in any organ, and further exerted anti-inflammatory and antitumorigenic effects resulting in increase of survival of Wistar Hannover rats.

## Introduction

Propolis, a honey bee hive product, is thought to exhibit a broad spectrum of biological activities including antimicrobial, antioxidant, anti-inflammatory, anti-allergic, dermatoprotective, laxative, antidiabetic, immunomodulatory and antitumor [1]. Some of them were attributed to artepillin C (3,5-diprenyl-4-hydroxycinnamic acid), caffeic acid (cinnamic acid) phenethyl esters (CAPE), baccharin, drupanin, chrysin, nemorosone, galangin, cardanol and other ingredients with oxyradical scavenging propertieswhich are involved in induction of cell-cycle arrest and apoptosis, suppression of matrix metalloproteinases, anti-angiogenesis, prevention of metastasis and moderation of the side effects induced by chemotherapy [1–9]. Propolis has been found to exert a protective effect *in vitro* and *in vivo* against breast, liver, pancreas, kidney, bladder, prostate, colon, brain, head and neck, skin and blood cancers, however, there is a lack of information about its clinical effectiveness [1, 10–14]. Chemical compounds of propolis target numerous biochemical and genetic pathways of cancer progression, however, its biological activities vary depending on the geographical origin of the botanical source and on the method of preparation [1]. Oral administration of Brazilian green propolis and its main constituent artepillin C was shown to suppress renal and pulmonary carcinogenesis due to its ability to inhibit lipid peroxidation [15]. Furthermore, inhibitory effect of aqueous and ethanol extracts of propolis on colon carcinogenesis was reported in rats [16, 17]. It has been shown to be nontoxic in humans or mammals unless very large quantities are administered [18]. Some of Brazilian propolis constituent flavones, such as quercetin, were found to be mutagenic by the Ames test, however, some authors reported no mutagenicity or even antimutagenic effects of propolis itself [19–24].

On the other hand, super critical carbon dioxide extract of Brazilian green propolis was shown to induce glutathione-S transferase placental form (GST-P) positive foci in the liver, and papillary or nodular hyperplasia (PN hyperplasia) in the urinary bladder when applied in a two-stage carcinogenesis model in F344 rats [25]. Furthermore, ethanol-extracted Brazilian green propolis (EEP) exerted promoting effect on F344 rat bladder carcinogenesis initiated with N-butyl-N-(4-hydroxybutyl) nitrosamine (BBN) [23]. Moreover, it was recently found to exert estrogenic effect in ovariectomized rats, significantly increasing uterine luminal epithelium thickness and ductal cell proliferation in the mammary glands [26]. Therefore, there is a doubt concerning propolis carcinogenicity in the liver, bladder, mammary gland and uterus.

So far, as the traditional use of Brazilian green propolis does not necessarily guarantee its safety [27], the 2-year rat study reported herein was undertaken in order to evaluate the chronic toxicity/carcinogenicity potential of EEP at doses of 0.5 and 2.5%, incorporated in the diet of male and female Wistar Hannover rats. All tissues and organs underwent the complete histopathological examination, furthermore, blood hematology and biochemistry analyses were done. Daily observation for growth and general health, and sequential evaluation of diet and water intakes were also performed to identify possible toxicity.

## Materials and Methods

The present study was approved by the Ethics Committee of the Institutional Animal Care and Use Committee of Osaka City University Graduate School of Medicine, Osaka, Japan (No. 04AI). Guidelines set by the National Institute of Health and Public Health Service Policy on the Humane Use and Care of Laboratory Animals were followed all the time. We conducted this study with reference to the Organization for Economic Co-operation and Development (OECD) Guideline for Testing Chemicals; and in accordance with the OECD principles of “Carcinogenicity Studies” (Test No. 451, 2009, pages 1–15) [28] and Good Laboratory Practice (GLP) (OECD 2009) [29].

### Test material and diets

Brazilian green propolis (the source plant: *Baccharis dracunculifolia*) powder used in the present study was manufactured by API Chemical Industry Co., Ltd. (Gifu, Japan). Propolis powder was prepared from the raw Brazilian propolis dissolved in ethanol. Impurities from pollen were eliminated by centrifugation and then ethanol was removed. The final product (EEP-B50P; Lot No.031204) was a yellow fine powder, water-soluble, light- and heat-stable. To detect the stability of the EEP, *p*-coumaric acid, artepillin C, baccharin and drupanin, the derivatives which are proposed to exhibit anticancer activity, were measured using HPLC600 (Waters, USA) with Millenium32-J system software and Capcell Pak ACR column at UV 300nm before the beginning and at the end of the test material administration, and confirmed to be acceptable by comparison of the data from two time points, with no significant difference being evident. Furthermore, the EEP sample composition in the diet was analyzed by HPLC. To prepare samples for the HPLC analysis, 30 ml of 5% EEP was added to the 10 g of MF powder diet and mixed. Four hours later the extraction was performed, and the obtained sample was applied onto HPLC. Artepillin C, *p*-coumaric acid, baccharin and drupanin at doses of 1 mg/ml and 0.5 mg/ml each were used as positive controls. According to the results of HPLC, the sample contained 7.40 W/W% artepillin C, 1.51 W/W% *p*-coumaric acid, 1.31 W/W% drupanin and 0.46 W/W% baccharin (S1 Fig). Finally dextrin powder (powderization substance) was added to Brazilian propolis powder to get the approximate ratio of 1:1. In the present experiment, the test diet powders were prepared as followers: 2.5% EEP diet contained 2.5% Brazilian propolis and 2.5% dextrin, and 0.5% EEP diet contained 0.5% Brazilian propolis and 2.5% dextrin in MF powder diet. The accuracy of doses formulation and uniformity of blending of the diets was performed by the analytical chemistry laboratories at Oriental Yeast Co., Tokyo, Japan. The ultraviolet spectroscopy and HPLC analyses were used to check the homogeneity and stability of dose formulations after the diets were stored for at least 2 weeks at room temperature (22–25°C).

### Animal husbandry and treatment

Wistar Hannover (BrlHan: WIST@Jcl (GALAS); LOT No. A730502-40-30) rats of both sexes were obtained at the age of 5 weeks from Clear Co. (Japan). Animals were housed in plastic cages (3 rats/cage) with wood chips for bedding, and allowed to acclimate for 1 week, then divided by stratified randomization into 3 male and 3 female body weight-matched groups, each comprising 50 rats. The environment was maintained at a temperature of 24±2°C and a relative humidity of 60±10% with lighting supplied with a 12-h light/dark cycle. All rats were given tap water *ad libitum*. During the experiment, all animals were kept in the SPF zone of the animal house, and all the conditions were strictly controlled, including the sterilization and autoclaving procedures, usage of the disposable plastic ware and sterilized reagents. The Brazilian propolis diets containing 0.5% and 2.5% EEP were prepared as described above and administered starting at 6 weeks of age for 104 weeks. Fifty rats of both sexes, serving as respective controls, were handled in the same manner as the propolis-exposed groups, but were fed the basal MF powder diet. All animals were checked for general behavior and signs of toxicity or moribund state once a day. Rats were carefully observed for 104 weeks. Body weights, food and water intakes were measured every week for the first 12 weeks, and every 4 weeks thereafter. During the experiment, the specific signs used to determine when the animal should be euthanized included the comatose condition or no response to stimuli, changes in external physical appearance and heart rate, dyspnea or severe breathing problem, hypothermia, prostration, body weight loss and related changes in food and water consumption. If the significant body weight loss or the food and water intakes changes were firstly detected, animal was checked more precisely for other signs of sickness, pain or moribund state. Rats were euthanized, and the systemic macroscopic pathological examination was performed. Wistar Hannover rats were found to be healthy and usually long-lived. Therefore, fortunately, we did not observe such condition as an accidental death.

In the preliminary 1-year toxicity study, propolis was administered to male and female rats at concentrations of 0% (control), 0.5% and 2.5%. No mortality was detected in any group. No significant decreases of body weights, changes in water consumption, and increases of liver or kidneys weights were evident for both sexes. In blood biochemistry, protein levels and parameters related to lipids or urea nitrogen were not altered. No increase of urinary ketones in the 2.5% EEP-treated males, or the yellow coloration of bones in both sexes was obvious. Furthermore, no histopathologicaly significant changes were found in all organs. Based on these findings, the assessment of possible carcinogenicity with long-term administration appeared pertinent, the concentration of 2.5% was estimated to be the maximum tolerated dose (MTD) and was selected as the highest dose for the present 2-year study. The lowest dose was set at 0.5%.

### Clinical observations and histopathological examination

Animals were observed daily for clinical signs. All rats underwent complete necropsy. Organs were removed, weighed, and examined for macroscopic lesions. The histopathological analysis of organs and tissues designated in the OECD test guidelines was performed for all rats. After fixation in 10% neutral buffered formalin and embedding in paraffin, tissue sections of 4 μm in thickness were prepared and stained with hematoxylin and eosin (H & E). The incidences of histopathological lesions, neoplastic, preneoplastic (proliferative) or non-neoplastic, were evaluated for all organs and tissues. In case of animals euthanized before the final necropsy, besides the gross masses, the complete histopathological analysis was done. The results were presented as selected organ tumor incidence for the effective number of rats.

### Hematology and blood biochemistry analyses

Blood was collected via the abdominal aorta from all survived rats at the end of the study after the overnight fasting. Blood biochemistry was performed in 10 rats per group per sex. The automated hematology analyzer (Sysmex XE-2100, Mitsubishi Chemical Visuals, Osaka, Japan) was applied for the hematological analysis of blood serum to detect the white and red blood cell counts, hemoglobin (Hb) and hematocrit (Ht) concentrations, mean corpuscular volume (MCV), mean corpuscular hemoglobin (MCH), mean corpuscular hemoglobin concentration (MCHC), platelet count, neutrophils, band neutrophils (stab cells), eosinophils, basophils, monocytes and lymphocytes counts. Biochemical analysis was performed using the automatic analyzer (Olympus AJ-5200, Tokyo, Japan) to determine the levels of total protein (T-protein, g/dL), albumin (g/dL), albumin/globulin ratio (A/G ratio), total bilirubin (T-bil, mg/dL), aspartate aminotransferase (AST, IU/L), alanine aminotransferase (ALT, IU/L), alkaline phosphatase (ALP, IU/L), γ-glutamyl transpeptidase (γ-GTP, IU/L), triglycerides (TG, mg/dL), total cholesterol (T-chol, mg/dL), blood urea nitrogen (BUN, mg/dL), creatinine (mg/dL), sodium (Na), chloride (Cl), potassium (K), calcium (Ca) and inorganic phosphorus (IP) (mEq/L).

### Statistical analysis

The significance of differences for each parameter (excluding general conditions) was analyzed using the StatLight-2000(C) program (Yukms corp., Japan) and the GraphPad Prism 5 Software Inc. (CA, USA). The significance of intergroup differences of incidences from gross pathology was analyzed using the Fisher’s exact probability test or the *χ*^2^-test. Differences in survival were analyzed by Kaplan-Meier method. Statistical comparisons of the numerical data among the control, high and low dose EEP groups were conducted using the Bartlett’s test. If homogeneous, the data were analyzed with the Dunnett’s multiple comparison test (two-sided), and if not, with the Steel’s test (two-sided). In all cases, a *p* value of 0.05 was considered to be significant.

## Results

### Survival, clinical signs, body and organ weights

**Fig 1:**
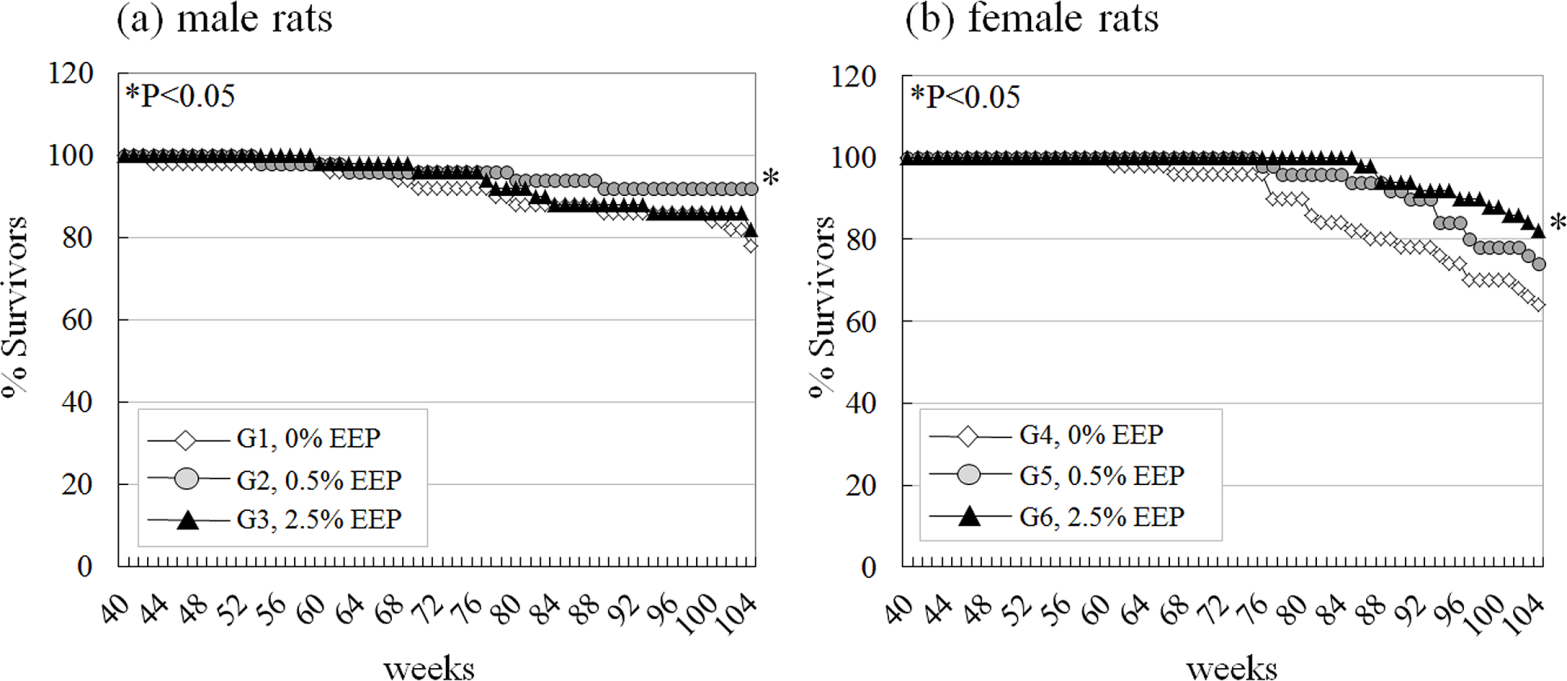
Survival curves of male and female rats fed 0.5% and 2.5% EEP-containing diet for 2 years.

Concentrations of EEP in diets were maintained constant throughout the 2-year exposure period. The survival rates of animals at the end of the study and survival curves are shown in Table 1 and Fig 1, respectively. Eleven, 4 and 9 male and 18, 13 and 8 female rats from 0%, 0.5% and 2.5% EEP groups, respectively, were found in moribund state at weeks 43–104. The main causes of mortality were pituitary tumors and lymphoma/leukemia. The other causes included thymomas, malignant mesotheliomas, mammary and uterine tumors. Significant increase of survival rate (P<0.05) was found in males of the 0.5% EEP group, attributable to a decreased number of deaths due to low incidences of pituitary tumors and lymphoma/leukemia. In females, the survival rates in 0.5% and 2.5% EEP groups were higher than in the control group for the same reasons, with the significant difference observed for 2.5% EEP-treated rats (P<0.05). Thus, mortality caused by pituitary tumors and lymphoma/leukemia in the 0.5% EEP male (pituitary tumors: 0 rats; lymphoma/leukemia: 3 rats) and 2.5% EEP female (pituitary tumor: 4 rats; lymphoma/leukemia: 0 rats) groups were less frequent than in the control groups (pituitary tumors: 2 male and 12 female rats; lymphoma/leukemia: 6 male and 4 female rats). Body weights in males of the 2.5% EEP group were slightly lower than in the control group, starting from week 10 and all throughout the study, but without significance (Table 1 and S2 Fig). Body weight in 2.5% EEP female group started to decrease from week 10, with significant difference from control group reached at the end of the experiment (Table 1). Final body weight was 80% (statistically significant) and 98% of control for 2.5% EEP-treated females and males, respectively. However, water and food consumptions for exposed rats of both sexes showed no variations among the groups. Furthermore, no significant changes of absolute or relative organ weights were noted in all treated groups (Table 1).

**Table 1:** Final survival rates, total EEP intake, final body and relative organ weights of Wistar Hannover rats.

		Male			Female	
Group	G1	G2	G3	G4	G5	G6
EEP (%)	0	0.5	2.5	0	0.5	2.5
No. of rats examined	50	50	50	50	50	50
No. of surviving rats^a^ (%)	39(78)	46(92)*	41(82)	32(64)	37(74)	42(84)*
Final body weight^b^ (g)	578.3±90.2	597.3±67.3	566.3±91.1	409.5±77.3	402.9±79.0	325.0±69.4*
Total EEP intake (mg/kg b.w.)	0	209008.8	1066811.2	0	258367.2	1176011.2
Organ weights						
Liver (g)	16.3±3.6	16.1±2.9	16.3±3.0	11.03±2.8	10.7±3.4	9.1±1.7
Liver (%)	2.83±0.38	2.69±0.35	2.83±0.32	2.67±0.41	2.64±0.61	2.85±0.40
Kidney (R.) (g)	1.69±0.53	1.58±0.32	1.68±0.34	1.12±0.17	1.17±0.15	1.00±0.16
Kidney (R.) (%)	0.30±0.09	0.27±0.05	0.29±0.05	0.28±0.05	0.30±0.07	0.32±0.06
Kidney (L.) (g)	1.65±0.43	1.51±0.36	1.84±1.21	1.07±0.16	1.08±0.23	0.97±0.16
Kidney (L.) (%)	0.29±0.07	0.25±0.06	0.32±0.18	0.27±0.04	0.27±0.08	0.31±0.06
Spleen (g)	1.24±0.35	1.22±0.35	1.09±0.32	0.87±0.46	0.93±0.31	0.71±0.29
Spleen (%)	0.22±0.05	0.20±0.05	0.19±0.04	0.21±0.09	0.23±0.08	0.22±0.09
Thymus^c^ (g)	0.151±0.123	0.148±0.142	0.137±0.111	0.147±0.118	0.101±0.052	0.093±0.040
Thymus (%)	0.025±0.019	0.024±0.022	0.024±0.018	0.037±0.029	0.025±0.014	0.029±0.011

Data are Mean ± SD for the surviving animals at the end of the 2-year administration period

Relative organ weights were calculated for surviving animals at termination of experiment with the following equation: Absolute organ weight/fasted body weight x 100

^a^ No. of surviving animals at the end of the 2-year administration period

^b^ Mean ± SD for body weights of the surviving animals at the end of the 2-year administration period

^c^ Thymus weights were calculated for animals without thymomas.

* Significantly different from the control groups at P<0.05

No significant differences of organ weights were observed as compared to the respective controls.

### Average food, water and EEP intakes

Final average food and water intakes of treated rats were similar to that of the corresponding controls (S3 and S4 Figs). EEP doses used in the present study were 0.5% (ranging between 200 and 400 mg/kg b.w./day for males and females, respectively) and 2.5% (ranging between 1000 and 2000 mg/kg b.w./day for males and females, respectively) consumed by a rat with body weight 500 g (male) and 300 g (female) in about 20 g of diet. It would be approximately equal to 2–4 and 10–20 mg/kg b.w./day intakes by a human with a mean body weight of 50 kg (the accepted WHO safety factor in terms of accepted dietary intake (ADI) for rats is 100). Dietary concentrations of 0.5% and 2.5% EEP applied to rats resulted in average daily intakes of 287.1 and 1465.4 mg/kg b.w./day for males, and 354.9 and 1615.4 mg/kg b.w./day for females, respectively. The total calculated EEP intakes were 209008.8 and 1066811.2 mg/kg b.w. for males, and 258367.2 and 1176011.2 mg/kg b.w. for females in 0.5% and 2.5% EEP groups, respectively.

### Histopathology

#### Effect of EEP on development of neoplastic and preneoplastic lesions

No treatment-related significant increase of tumor, preneoplastic or proliferative lesions incidences was found in any organ or tissue of rats administered EEP. All data were within the normal range of tumor incidences seen in Wistar Hannover rats after 2 years accordingly to the data provided by Clea Japan, Inc.

Data for incidences of lymphoma/leukemia and neoplastic (benign and malignant) and preneoplastic (proliferative) lesions in the liver, kidneys, thymus, pituitary gland, thyroid and adrenals of Wistar Hannover rats, which represented significant histopathological changes, are shown in Table 2. Significantly decreased incidences of pituitary tumors (pituitary adenoma and total tumors in 0.5% EEP male group, and carcinoma and total tumors in 2.5% EEP female group) were detected (Table 2). Furthermore, reduction of incidence of lymphoma/leukemia in a concentration-dependent manner was apparent, with significance at 2.5% EEP in males and females, along with the significant decrease in incidences of lymphoid hyperplasia (HPL) of thymus, mandibular and mesenteric lymph nodes in the 2.5% EEP-treated animals (Table 2). In addition, incidences of total thyroid gland tumors in males at a dose of 0.5% and adrenals cortical hyperplasia in females at 0.5 and 2.5% EEP dose levels were significantly lower as compared to the controls (Table 2).

**Table 2:** Incidences of lymphoma/leukemia and neoplastic (benign), neoplasmic (malignant) and preneoplastic (proliferative) lesions in the liver, kidneys, thymus, pituitary gland, thyroid and adrenals of Wistar Hannover rats administered EEP for 2 years.

Incidence (No. rats (%))		Male			Female	
Group	G1	G2	G3	G4	G5	G6
EEP (%)	0	0.5	2.5	0	0.5	2.5
No. of rats examined	50	50	50	50	50	50
Scheduled sacrifice	39	46	41	32	37	42
Unscheduled sacrifice	11	4	9	18	13	8
Lymphoma/leukemia	8(16)	4(8)^a^	1(2)***	3(6)	1(2)	0**
Mandibular lymph nodes lymphoid HPL	24(48)	15(30)	9(18)**	18(36)	14(28)	7(14)**
Mesenteric lymph nodes lymphoid HPL	13(26)	10(20)	7(14)*	20(40)	15(30)	8(16)**
Thymus						
Lymphoid HPL	10(20)	8(16)	3(6)***	9(18)	2(4)	2(4)**
Thymoma	3(6)	2(4)	2(4)	3(6)	2(4)	2(4)
Liver						
Clear cell foci of cellular alteration	13(26)	14(28)	9(18)	3(6)	0	2(4)
Eosinophilic foci of cellular alteration	2(4)	1(2)	2(4)	1(2)	0	0
Basophilic foci of cellular alteration	2(4)	0	0	0	0	0
Fatty metamorphosis	6(12)	1(2)	1(2)	6(12)	6(12)	1(2)
Fibrosis	7(14)	2(4)	0	5(10)	6(12)	1(2)
Hepatocellular adenoma	0	1(2)	0	0	1(2)	0
Kidneys						
Tubular HPL	25(50)	8(16)**	5(10)**	7(14)	3(6)	2(4)**
Adenoma	2(4)	0	0	0	0	0
Liposarcoma	0	0	1(2)	0	0	0
Total tumors	2(4)	0	1(2)	0	0	0
Pituitary gland						
Adenoma (anterior lobe)	10(20)	2(4)*	6(12)	18(36)	15(30)	10(20)
Carcinoma	1(2)	0	0	8(16)	3(6)	0**
Total tumors	11(22)	2(4)**	6(12)	26(52)	18(36)	10(20)**
Thyroid						
C-cell HPL	16(32)	9(18)	14(28)	10(20)	11(22)	5(10)
Follicular cell HPL	1(2)	2(4)	1(2)	2(4)	2(4)	0
C-cell adenoma	1(4)	0	0	1(2)	1(2)	2(4)
C-cell carcinoma	3(6)	0	0	1(2)	0	1(2)
Follicular cell adenoma	0	0	1(2)	0	1(2)	1(2)
Follicular cell carcinoma	1(2)	0	1(2)	1(2)	1(2)	0
Total tumors	5(10)	0*	2(4)	3(6)	3(6)	4(8)
Adrenals						
Cortical HPL	3(6)	2(4)	2(4)	26(52)	13(26)*	9(18)**
Cortical adenoma	0	0	1(2)	1(2)	2(4)	0
Cortical carcinoma	1(2)	0	0	0	0	0
Total tumors	1(2)	0	1(2)	1(2)	2(4)	0

*P<0.05

**P<0.01

***P<0.001: significantly different v.s. male or female control diet group.

^a^P = 0.05

HPL, hyperplasia.

Data for incidences of neoplastic lesions in other organs or tissues besides the lymphoma/leukemia, liver, kidneys, thymus, pituitary gland, thyroid and adrenals, which did not represent significant changes, are shown in Table 3. Despite higher incidences of uterine adenocarcinoma, leiomyosarcoma and endometrial stromal polyps in 2.5% EEP female group, the total incidences of uterine epithelial and non-epithelial lesions in females in high dose group did not reach statistical significance as compared to the female control group. Therefore, it was judged that the incidence of uterine tumors was not significantly affected by administration of EEP. Furthermore, trends for decrease of incidences of islet cell pancreas adenoma in males and mammary fibroadenoma in females were also observed in EEP-treated rats.

**Table 3:** Incidence of neoplastic lesions observed in the other organs or tissues of Wistar Hannover rats administered EEP for 2 years.

Incidence (No. rats (%))		Male			Female	
Group	G1	G2	G3	G4	G5	G6
EEP (%)	0	0.5	2.5	0	0.5	2.5
No. of rats examines	50	50	50	50	50	50
Lung(s)						
Adenoma	1(2)	0	0	0	0	0
Pancreas						
Islet-cell adenoma	3(6)	1(2)	1(2)	1(2)	0	1(2)
Abdominal cavity						
Mesothelioma	2(4)	0	2(4)		0	1(2)
Skin/subcutis						
Fibrosarcoma	3(6)	2(4)	1(2)		0	0
Lipoma	0	0	0		1(2)	0
Squamous cell carcinoma	0	1(2)	1(2)		0	0
Total	3(6)	3(6)	2(4)		1(2)	0
Mammary gland						
Fibroadenoma	0	0	0	8(16)	6(12)	4(8)
Fibroma	0	0	0	1(2)	1(2)	0
Adenoma	0	0	2(4)	0	0	0
Adenolipoma	0	0	0	0	1(2)	0
Adenocarcinoma	0	0	0	3(6)	7(14)	3(6)
Total	0	0	2(4)	12(24)	15(30)	7(14)
Uterus						
Epithelial lesions						
Adenoma				1(2)	1(2)	1(2)
Adenocarcinoma				0	1(2)	4(8)
Squamous cell carcinoma				1(2)	0	1(2)
Total				2(4)	2(4)	6(12)
Non-epithelial lesions						
Leiomyosarcoma				1(2)	0	2(4)
Endometrial stromal polyp				8(16)	8(16)	10(20)
Total				9(18)	8(16)	12(24)

Since a previous report on EEP documented its promoting effect in the bladder and liver of F344 rats [23, 25], special attention was given to examination of bladders and livers in all groups. Importantly, no increase of tumor or preneoplastic lesions incidences was indicated in the liver of EEP-treated Wistar Hannover rats. Furthermore, there were no signs of development of neoplasms in the bladder including adenomas, carcinomas or PN hyperplasia. Occasional tumors in other sites such as respiratory system, skin/subcutis, abdominal cavity sex organs and brain, were within the normal range of aged rats and were comparable among the groups.

### Effect of EEP on development of non-neoplastic lesions

In general, the non-neoplastic lesions were mainly related to aging and common for this strain of rats after 2 years. Significant inhibition of chronic kidney nephropathy was indicated in the high dose EEP-treated male and female rats (22% in males and 14% in females) as compared to the control groups (56% in males and 38% in females). Moreover, incidences of tubular HPL in kidneys exhibited a significantly negative trend in 0.5% and 2.5% EEP-treated male and 2.5% EEP-treated female rats as compared with respective control groups (Table 2). Furthermore, levels of lymphocytic accumulations, fibrosis in the liver and kidneys and fatty metamorphosis in the liver showed dose-dependent trends for decrease in animals administered EEP (Table 2). In addition, a significantly decreased incidence of cortical HPL in adrenals was found at 0.5% and 2.5% EEP-treated female rats (Table 2).

### Hematology and blood biochemistry

No significant changes in hematological parameters were observed in any EEP-treated group of either sex, except for significant decreases in mean neutrophil and band neutrophil (stab cells) counts in 0.5 and 2.5% EEP-treated males and 2.5% EEP-treated females, respectively, being indicative of suppressed inflammation (Table 4).

**Table 4:** Complete blood counts, hematology and biochemistry data.

		Male			Female	
Group	G1	G2	G3	G4	G5	G6
EEP (%)	0	0.5	2.5	0	0.5	2.5
No. of rats examined (hematology/biochemistry)	39/10	46/10	41/10	32/10	37/10	42/10
WBC (/μl)	3821±2122	3602±2952	3224±1644	2310±1028	2960±2550	3290±2432
RBC (x10^4^/μl)	785.0±50.6	801.0±47.7	805.6±34.4	714.8±75.2	705.1±84.5	680.9±120.8
Hb (g/dl)	14.2±0.9	14.3±0.8	14.4±0.5	13.9±1.5	13.6±1.5	12.8±2.1
Ht (%)	43.6±2.7	43.9±2.7	43.8±1.6	43.0±3.8	42.3±4.2	39.7±6.1
MCV (fl)	55.5±2.7	54.8±2.2	54.4±2.5	60.4±3.3	60.1±3.0	59.1±5.5
MCH (pg)	18.1±0.7	17.8±0.6	17.8±0.7	19.5±0.7	19.4±0.9	18.9±1.6
MCHC (g/dL)	32.6±0.9	32.5±1.1	32.8±0.8	32.3±1.4	32.2±1.0	32.0±.58.0
Platelets (x10^10^/L)	84.1±12.7	82.7±17.4	81.7±9.6	73.1±11.1	71.7±19.2	78.6±33.5
Neutrophils (x10^3^/L)	36.3±15.5	29.3±10.2*	27.9±11.0**	36.3±10.5	33.4±15.1	34.2±15.3
Band neutrophils (x10^3^/L)	1.0±0.8	0.7±0.7	0.7±0.6	0.9±0.7	0.9±0.6	0.6±0.6*
Eosinophils (x10^3^/L)	1.2±1.0	1.1±1.1	1.4±1.2	1.1±1.2	1.3±1.9	1.0±1.0
Basophils (x10^3^/L)	0.00±0.00	0.00±0.00	0.02± 0.16	0.00±0.00	0.00±0.00	0.02±0.16
Monocytes (x10^3^/L)	2.7±1.4	2.1±1.4	1.7±1.4	1.8±0.9	2.0±1.8	1.7±1.2
Lymphocytes (x10^3^/L)	58.8±15.8	66.8±10.7	68.3±11.6	59.8±10.5	62.4±16.7	60.2±19.1
T-protein (g/dl)	6.9±0.5	6.7±0.4	6.6±0.2	6.9±0.3	6.8±0.5	6.9±0.7
Albumin (g/dL)	3.0±0.2	2.9±0.1	3.1±0.1	3.2±0.3	3.1±0.3	3.4±0.4
A/G ratio	0.8±0.1	0.8±0.1	0.9±0.1	0.9±0.1	0.8±0.1	1.0±0.1
T-BiL (mg/dL)	0.2±0.0	0.2±0.0	0.2±0.0	0.2±0.0	0.2±0.0	0.2±0.0
AST (IU/L)	174.3±103.6	118.8±44.1	103.9±31.5^a^	98.1±16.5	121.7±35.5	148.1±137.6
ALT (IU/L)	223.1±363.3	40.2±17.6	34.4±19.2	35.0±11.7	39.5±19.9	55.5±83.7
ALP (IU/L)	249.6±126.2	194.8±78.3	213.1±104.8	104.5±64.5	124.2±47.3	159.7±178.0
γ-GTP (IU/L)	1.0±0.0	1.2±0.6	1.6±0.7	2.3±3.5	1.0±0.0	1.3±1.0
T-Cholesterol (mg/dL)	161.2±71.1	138.4±49.9	115.5±54.3	96.2±36.1	95.4±19.0	73.5±18.5*
TG (mg/dL)	241.2±154.9	174.8±58.9	205.6±168.1	353.8±477.2	131.0±75.1	57.5±27.4*
BUN (mg/dL)	16.3±2.8	16.5±2.1	18.2±1.0	16.9±3.7	17.4±4.7	18.6±4.0
Creatinine (mg/dL)	0.4±0.1	0.3±0.0	0.3±0.1	0.3±0.1	0.3±0.0	0.3±0.1
Na (mEq/L)	145.0±1.3	143.8±1.2	145.3±1.6	142.6±2.1	142.6±1.9	142.1±1.9
K (mEq/L)	4.7±1.5	4.4±0.3	4.5±0.2	4.1±0.6	4.0±0.3	4.8±1.5
Cl (mEq/L)	100.8±3.5	101.8±3.3	103.7±1.8	100.6±2.5	99.1±3.3	101.1±3.0
Ca (mEq/L)	10.9±0.5	10.7±0.3	10.7±0.2	10.8±0.4	10.7±0.4	10.6±0.4
IP (mEq/L)	6.1±1.7	5.2±0.7	5.6±0.6	5.1±0.7	5.6±0.8	5.5±1.7

Values are means ± SD

*P < 0.05

**P<0.01

^a^P = 0.05

TG, triglycerides; T-Bil, T-bilirubin

IP, inorganic phosphorus.

In blood biochemistry, a significant reduction and trends for decrease of total cholesterol and triglycerides levels were found in 2.5% EEP-treated females and 0.5 and 2.5% EEP-treated males, respectively (Table 4). In addition, the dose-dependent trends for decrease were detected for AST and ALT levels in the blood of male rats administered EEP.

## Discussion

In the present 2-year feeding study, overall histopathological evaluation of neoplasms in all tissues showed no significant increase of tumor incidence in any organ or tissue of 0.5% and 2.5% EEP-treated male and female Wistar Hannover rats. The incidences of non-neoplastic findings among rats examined for toxicity were also mainly unaffected by the EEP treatment, furthermore, no significant changes of relative organ weights were found in both males and females. In addition, clinical condition of rats was unaffected.

Significant increases in survival rates of 0.5% and 2.5% EEP-treated males and females, respectively, were observed, which were considered to be causally related to the EEP exposure, on the basis of the following evidence. First, the incidences of pituitary tumors in 0.5% and 2.5% EEP-treated male and female rats, respectively, were significantly lower than in respective control groups, being in line with changes of survival. Second, malignant lymphoma/leukemia incidences were significantly decreased in an exposure concentration-dependent manner in both males and females administered EEP. Furthermore, incidence of total thyroid tumors in males of the 0.5% EEP-exposed group was significantly lower as compared to the control. Importantly, there were no apparent effects of EEP on development of liver or bladder neoplasms and preneoplastic lesions.

In previous studies, increase of incidence of PN hyperplasia but not tumors and elevation of the number and area of GST-P-positive foci by 0.1% supercritical extract of Brazilian green propolis in the urinary bladder and liver, respectively, was shown using a two-stage carcinogenesis model (DMBDD) in F344 rats [25]. Furthermore, recently, Xie et al. (2015) reported an increased incidence and multiplicity of urothelial carcinomas in the bladder of male rats exposed to EEP in the diet at doses 0.125 ~ 1% for 32 weeks following initiation with BBN. EEP has been proposed to enhance BBN-initiated F344 rat urinary bladder carcinogenesis in a non-genotoxic manner by increasing formation of urinary precipitate, enhancing cell proliferation and inhibiting apoptosis during the early stages of carcinogenesis. However, ethanol extracts of propolis have been found to be mutagenicity negative in the urinary bladder urothelium of *gpt* delta rats and further exert antimutagenic effects in a dose-dependent manner at concentrations 0.1–4% against two mutagenic substances, azide sodium and potassium permanganate, in the presence and the absence of microsomal homogenate of mouse liver [23, 24]. Therefore, whether the promotion effect of EEP observed in previous studies with urinary bladder can be extrapolated to humans is inconclusive at present since EEP appeared to be not genotoxic and lacks cancer initiation activity. Furthermore, here we found that it is likely to exert protective effect on tumorigenesis in Wistar Hannover rats, thus, EEP modifying effects are likely to be rat strain dependent. The main Brazilian green propolis constituents, artepillin C, *p*-coumaric acid, cinnamic acid, caffeic acid, ferulic acid, and their derivatives including baccharin and drupanin, are likely to be responsible for the activities of EEP detected in the present 2-year carcinogenicity test.

Previous studies have demonstrated that exposure to different concentrations of propolis did not produce a carcinogenic effect in peripheral human lymphocytes *in vitro* but from the ability of propolis to increase the micronucleus (MN) rates, it has been suggested that it could exert carcinogenic effect at high concentrations [30]. Previously, no side effects were observed in mice, rats and humans after Brazilian propolis administration [31]. Nevertheless, there are cases of allergy and contact dermatitis reported [30]. Although results from some studies have demonstrated that it can act as promoter of liver and bladder carcinogenesis in rats at high doses, most investigations have reported anti-inflammatory, anti-viral or anti-allergic effects and protective roles in heart diseases, diabetes and cancer [30]. Propolis is considered nontoxic, and the safe concentration for humans is approximately 1.4 mg/kg b.w./day or 70 mg/day [30], what is close to the 0.5% dose of EEP applied in the present study. Propolis has low acute oral toxicity, as shown by the LD50 test in mice (2000 to 7300 mg/kg b.w.), and its flavonoids testing in rats (8000 to 4000 mg/kg b.w.) [32]. Moreover, no side effects have been seen in case of oral administration of EEP to mice in diet at concentrations higher than 4000 mg/kg b.w./day for two weeks, and treatment of mice and rats in their drinking water at 1400 mg/kg b.w./day for 90 days and 2740 mg/kg b.w./day for 60 days, respectively. Importantly, the present study demonstrated no carcinogenicity of EEP applied to Wistar Hannover rats for 2 years at a dose of 2.5% (1000–2000 mg/kg b.w./day).

Suppression of development of lymphoma/leukemia and pituitary tumors observed here is regarded as having relevance as antitumor effects of EEP. Furthermore, in the present study, we observed the clear anti-inflammatory and anti-proliferative activities of EEP in various organs of Wistar Hannover rats such as lymph nodes, thymus, adrenals and kidneys. Moreover, in hematology analysis, a significant decrease of neutrophils and band neutrophils numbers in EEP-treated rats pointed out the lower levels of inflammation. These results supported the recent reports, suggesting that antitumor effects of propolis could be attributed to its modulatory effect on immune system, which include macrophage activation, modulation of B, T lymphocytes, natural killer (NK) cells and antibody proliferation, production of cytokines (IL-2, IL-10 and IFN-gamma), downregulation of the toll-like receptor 2 (TLR-2) and human leukocyte antigen (HLA-DR) expression, induction of H_2_O_2_ release, inhibition of nitric oxide, prostaglandin and leukotriene generation, as well as suppression of the lipoxygenase pathway of arachidonic acid metabolism, myeloperoxidase activity, NADPH-oxidase ornithine decarboxylase, tyrosine-protein-kinase, hyaluronidase and downregulation of transcription factors [4, 33–35]. This anti-inflammatory activity can be explained by the presence of active flavonoids and cinnamic acid derivatives. The former includes quercetin, acacetin and naringenin; the latter includes baccharin, drupanin and CAPE [32]. Different components of Brazilian, Cuban and Mexican propolis, were found to exert pro- and anti-inflammatory effects depending on the dose, what may be useful for the development of novel immunomodulatory drugs [36].

Recently, propolis anti-angiogenic effects have been demonstrated, as well as augmentation of natural defense mechanisms such as tumor necrosis factor and caspase pathways, and increased apoptosis due to enhancement of Bax and TRAIL-R2 protein expression, activition of p38 MAP kinases and NF-kappaB and down-regulation of ERK 1/2 [30, 37–39]. Propolis and its chemical constituents artepillin C, baccharin and drupanin, CAPE and chrysin, exhibited potent cytotoxic effects and induced marked levels of apoptosis in breast, cervical, colon, intestine, liver, lung, prostate, skin cancers and leukemia in various animal and *in vitro* models [3, 6, 15, 37, 40, 41]. Furthermore, EEP anti-tumorigenic effects were also bound to its anti-proliferative activity in different carcinoma cells [10].

The antioxidant activity of propolis was considered to be the important factor for its proposed antitumor and hepatoprotective activity. Artepillin C and CAPE exhibited oxyradical scavenging properties and were shown to play a great role in propolis immunomodulatory effect [15, 30, 42–45]. Enhancement of endogenous antioxidant defenses by propolis was linked to the direct elimination of reactive oxygen species, inhibition of lipid peroxidation and oxidized glutathione level, increase of reduced glutathione level and restoration of activities of antioxidants enzymes, such as superoxide dismutase, catalase, glutathione S-transferase and glucose 6-phosphate dehydrogenase, activatiin of Erk-Nrf2-HO1, GCLM, and TrxR1 signal pathways [46, 47]. In support of our data demonstrating the decrease of AST and ALT levels in the blood induced by EEP, previously, the release of serum transaminases, alkaline phosphatase, lactate dehydrogenase, and γ-GTP was shown to be significantly restored with propolis treatment suggesting that it has a potential as a hepatoprotective agent [47].

In the present study, a significant decreases of female rat body weights, and T-cholesterol and triglicerides levels in the blood were observed, what was not a result of toxicity, but likely due to the estrogenic effect of EEP. There was a non-significant increase of uterine tumors incidence in high dose female group, and we concluded that in the present conditions, 2.5% EEP is likely to exert estrogenic activity but lacks the carcinogenicity in the uterus of Wistar Hannover rats. Previously, the estrogenic effects of propolis were demonstrated through the activation of an estrogen receptor [26]. For instance, EEP was found to bind human estrogen receptors and to induce the expression of estrogen-responsive genes in ER-positive MCF-7 and Ishikawa cells and to induce estrogenic activity in ER-expressing organs of ovariectomized rats. Moreover, selective binding of propolis to human estrogen receptor beta (but not alpha), with no estrogenic effect on estrogen receptor-positive breast cancer cells, has been demonstrated in female rats [48]. In addition, recent studies suggested that EEP has estrogen-like activity, possibly from isoflavones [49].

## Conclusions

Under the conditions of the 2-year feeding experiment, EEP did not exert carcinogenicity or induce significant histopathological changes in any organ, and further exerted anti-inflammatory and antitumorigenic effects in Wistar Hannover rats of either sex resulting in increase of survival.

## Supporting Information

S1 Fig The HPLC chromatogram of the ethanol-extracted Brazilian green propolis used in the present study. (TIF)

S2 Fig Body weight curves of Wistar Hannover rats. (TIF)

S3 Fig Food consumption curves of Wistar Hannover rats. (TIF)

S4 Fig Water consumption curves of Wistar Hannover rats. (TIF)
