# Supplementary figures and images for: Ethanol-Extracted Brazilian Propolis Exerts Protective Effects on Tumorigenesis in Wistar Hannover Rats
Source: PLoS One. 2016 Jul 8;11(7):e0158654. doi: 10.1371/journal.pone.0158654 (PMC4938237; doi:10.1371/journal.pone.0158654)

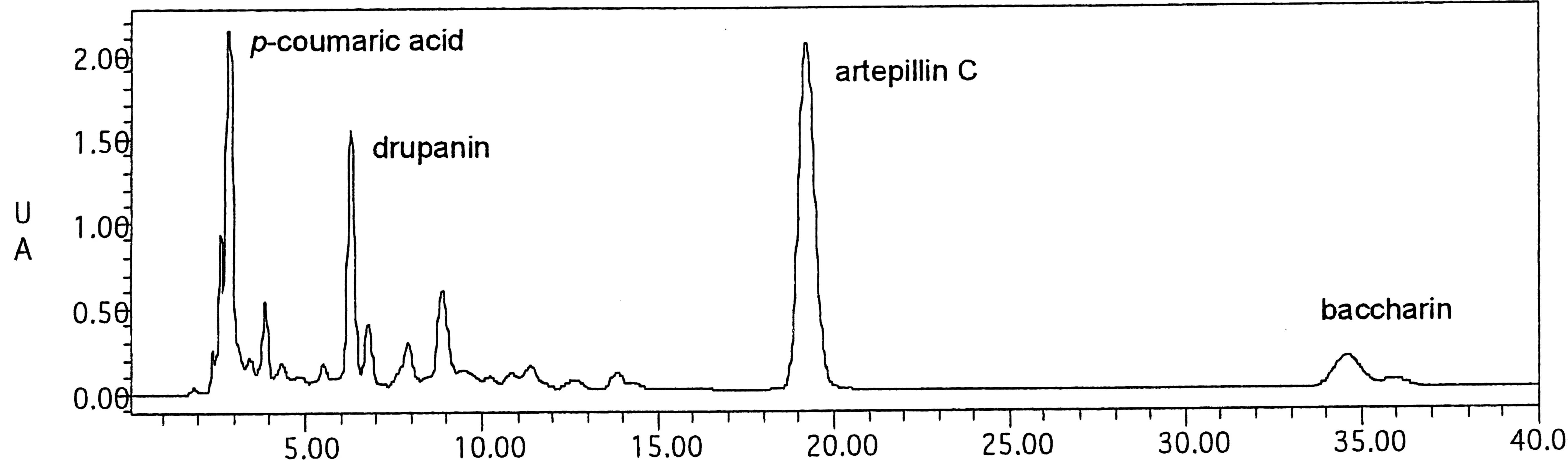

Supplement: S1 Fig — (TIF) [file pone.0158654.s001.tif]

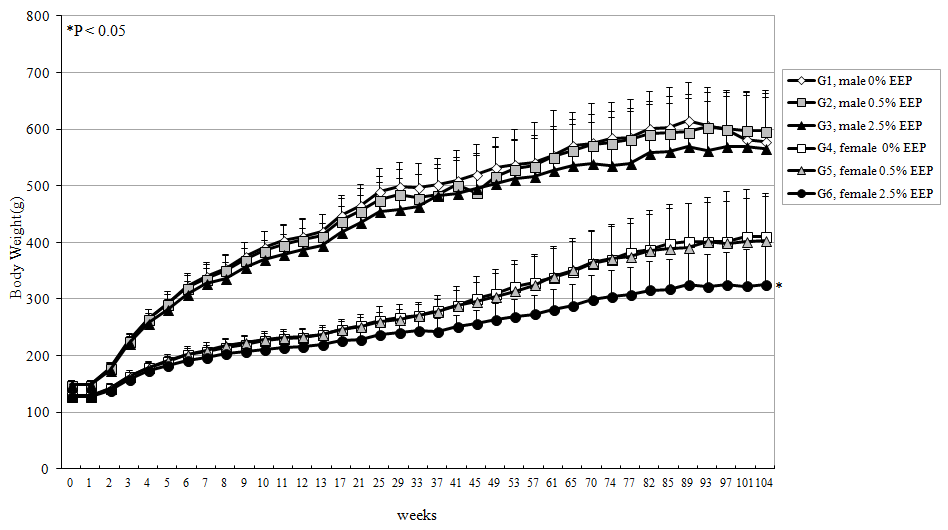

Supplement: S2 Fig — (TIF) [file pone.0158654.s002.tif]

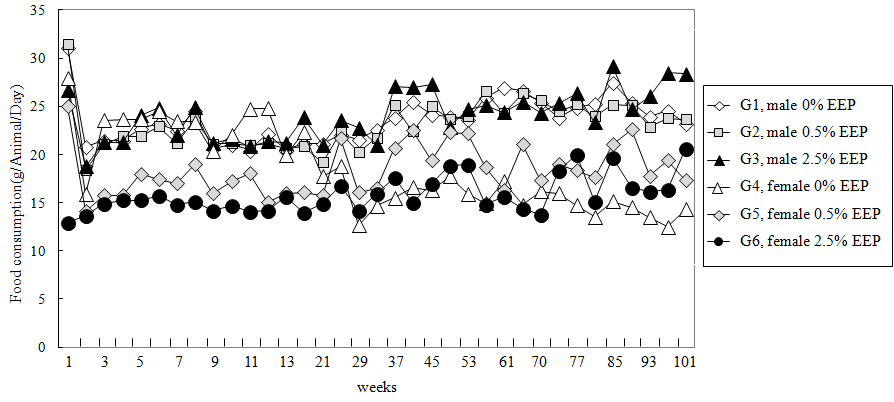

Supplement: S3 Fig — (TIF) [file pone.0158654.s003.tif]

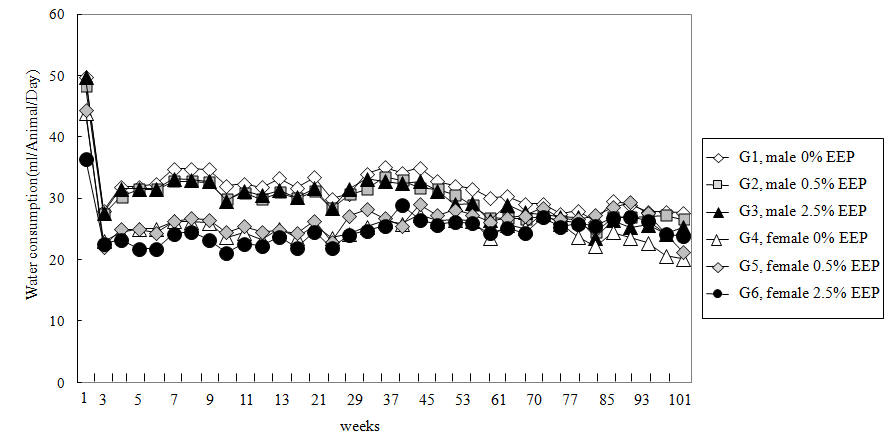

Supplement: S4 Fig — (TIF) [file pone.0158654.s004.tif]
